# Elucidating Iron Metabolism through Molecular Imaging

**DOI:** 10.3390/cimb46040175

**Published:** 2024-03-22

**Authors:** Feifei Liao, Wenwen Yang, Linzi Long, Ruotong Yu, Hua Qu, Yuxuan Peng, Jieming Lu, Chenghuan Ren, Yueqi Wang, Changgeng Fu

**Affiliations:** 1 Beijing University of Traditional Chinese Medicine Graduate School, Beijing University of Chinese Medicine, Beijing 100105, China; 20220931933@bucm.edu.cn (F.L.); 20230931939@bucm.edu.cn (R.Y.); 20210931914@bucm.edu.cn (Y.P.); 20220931934@bucm.edu.cn (J.L.); 20230931938@bucm.edu.cn (C.R.); 2 Graduate School, China Academy of Chinese Medical Sciences, Beijing 100091, China; werweny@163.com (W.Y.); qixiang830803@163.com (L.L.); hua_qu@yeah.net (H.Q.); 3 CAS Key Laboratory of Molecular Imaging, Beijing Key Laboratory of Molecular Imaging, The State Key Laboratory of Management and Control for Complex Systems, Institute of Automation, Chinese Academy of Sciences, Beijing 100190, China

**Keywords:** iron metabolism, molecular mechanism, molecular imaging technology, visualization, iron homeostasis

## Abstract

Iron is essential for many physiological processes, and the dysregulation of its metabolism is implicated in the pathogenesis of various diseases. Recent advances in iron metabolism research have revealed multiple complex pathways critical for maintaining iron homeostasis. Molecular imaging, an interdisciplinary imaging technique, has shown considerable promise in advancing research on iron metabolism. Here, we comprehensively review the multifaceted roles of iron at the cellular and systemic levels (along with the complex regulatory mechanisms of iron metabolism), elucidate appropriate imaging methods, and summarize their utility and fundamental principles in diagnosing and treating diseases related to iron metabolism. Utilizing molecular imaging technology to deeply understand the complexities of iron metabolism and its critical role in physiological and pathological processes offers new possibilities for early disease diagnosis, treatment monitoring, and the development of novel therapies. Despite technological limitations and the need to ensure the biological relevance and clinical applicability of imaging results, molecular imaging technology’s potential to reveal the iron metabolic process is unparalleled, providing new insights into the link between iron metabolism abnormalities and various diseases.

## 1. Introduction

Iron is one of the most abundant elements in the earth’s crust and serves as an indispensable trace element critical for the health and functioning of organisms. Iron level regulation is crucial; its excess and deficiency can lead to adverse health effects, necessitating sophisticated mechanisms for iron homeostasis. These regulatory processes are intricate, encompassing both systemic and intracellular dimensions. Systemically, iron regulation involves managing dietary intake, recirculation within the body, and losses. Intracellularly, it includes the mechanisms for iron absorption, storage, transport, and utilization [1]. The meticulous regulation of iron metabolism is pivotal for circumventing cytotoxicity associated with iron accumulation while ensuring that iron levels remain within a physiologically acceptable range to facilitate its crucial role as a catalytic component in numerous proteins and enzymes [2,3]. Recent advancements in iron metabolism have uncovered many intricate pathways pivotal to maintaining iron homeostasis [4,5]. Investigating the causes of imbalances in iron metabolism is essential to gain insights into diseases associated with iron dysregulation and to improve interventions.

Molecular imaging (MI) is an advanced imaging modality that combines imaging medicine, clinical medicine, molecular biology, molecular pathology, chemical physics, information science, etc. This technique is instrumental in characterizing and quantifying biological processes at the cellular and molecular levels within living organisms, including animals, cells, molecules, genes, and humans, as well as in various model systems [6,7]. By employing in vitro imaging detectors, MI empowers researchers to visualize and monitor iron dynamics at cellular and tissue levels in real time, thus providing vital information for the early diagnosis and treatment of relevant diseases.

The regulation of iron metabolism and the visualization of its processes through imaging techniques constitute pivotal fields of investigation. In this review, we delineate the essential functions of iron within biological systems and elucidate the molecular mechanisms governing iron metabolism in vivo. We further explore the significance of critical molecular pathways in the context of disease treatment and detection. Additionally, we provide a comprehensive summary of MI methodologies for visualizing iron metabolism processes, underscoring their critical role in advancing our understanding and management of related disorders.

## 2. The Vital Role of Iron in the Human Body

Iron is an essential trace element found in nearly all human body tissues, with healthy adults possessing a total iron content of 3 to 5 g. Over 70% of this iron is present in hemoglobin (Hb) and myoglobin (Mb), while approximately 25% is stored in the form of ferritin and hemosiderin in the liver, spleen, and bone marrow [8]. By binding with other proteins and organic groups, iron plays multiple crucial roles in vital processes, including oxygen transport, cellular respiration, immune function, DNA synthesis, and drug metabolism. Both deficiency and excess iron can lead to various disease states within the organism.

Firstly, iron, the cornerstone for Hb and Mb synthesis, plays a pivotal role in erythropoiesis and the efficient transport and storage of oxygen [9]. In this process, iron first participates in the synthesis of heme in the bone marrow hematopoietic cells. The heme then combines with globin to form Hb, ensuring the production of red blood cells and maintaining normal hematopoietic function in the body. Hemoglobin in red blood cells binds reversibly with oxygen, transporting it from the lungs to various tissues throughout the body via the bloodstream; myoglobin receives oxygen transported by Hb and stores it in muscle tissues to meet the body’s oxidative needs. An imbalance in iron intake, circulation, storage, and loss can lead to iron-deficient erythropoiesis in the bone marrow, subsequently causing anemia. The World Health Organization (WHO) estimates that approximately 40% of children aged 6–59 months, 37% of pregnant women, and 30% of women aged 15–49 worldwide suffer from anemia [10,11]. For athletes and individuals engaged in physical activity, adequate iron is crucial for optimal performance since myoglobin can rapidly supply energy [12,13].

As a crucial transition metal, iron is an indispensable cofactor in various enzyme-catalyzed reactions and is essential for regulating enzyme activity. Particularly in the functioning of cytochrome P450 enzymes (CYPs), iron directly influences the catalytic efficiency of CYPs through its involvement in the home center. Heme, formed by the complex of iron with a porphyrin ring, is crucial in ensuring that CYPs can effectively metabolize a wide range of endogenous and exogenous substances [14]. Moreover, iron also plays a role in other critical enzymes, like peroxidases, facilitating the transfer of electrons and protons through its transitions between different oxidation states (including Fe^2+^, Fe^3+^, and higher oxidation states), thereby supporting cellular energy metabolism and respiration. In summary, iron regulates the activity of many enzymes in various ways, influencing cellular energy metabolism and function and indirectly affecting the synthesis and repair of DNA.

Furthermore, the element iron plays a pivotal role in immune function. During the immune response, iron participates in intracellular metabolic activities through antioxidative reactions, mitochondrial oxidative phosphorylation, and inflammation regulation, enhancing the development and activation of immune cells like lymphocytes, macrophages, and granulocytes. It modulates the functions of these immune cells, ensuring the proper execution of immune functions. An iron deficiency may compromise the immune system, affecting the regulation and balance of immune responses, thereby reducing the body’s resistance to infections and diseases. Therefore, maintaining adequate levels of iron within the body is crucial for the optimal functioning of the immune system.

In summary, iron is an indispensable metallic element for maintaining life, and a constant balance between iron absorption, transportation, storage, and distribution is conducive to reducing disease development.

## 3. Molecular Mechanisms of Iron Metabolism In Vivo

**Figure 1:**
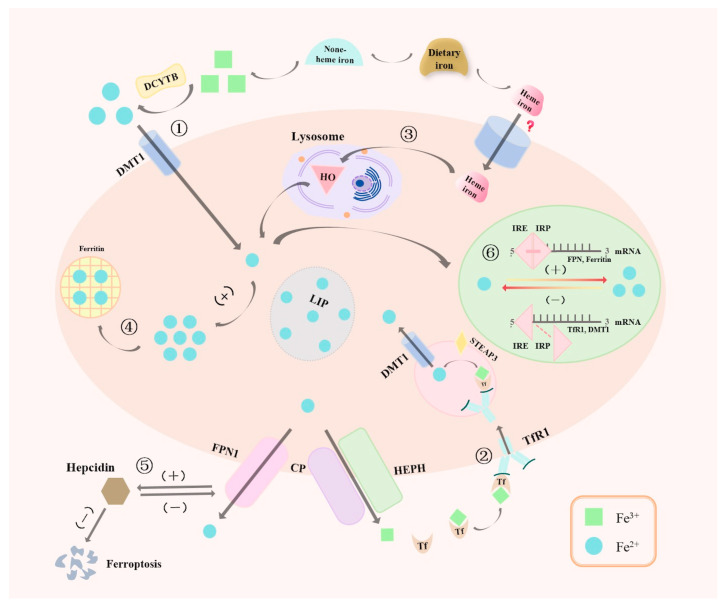
Iron homeostasis is highly regulated at both systemic and whole body. ① Non-heme iron is converted from Fe^3+^ to Fe^2+^ under the action of DCYTB. ② Tf carries Fe^3+^ into the cell via endocytosis through TfR1, where it is converted to Fe^2+^ under the action of STEAP3. ③ Heme iron releases Fe^2+^ in the lysosome through the action of HO. ④ Excess Fe^2+^ is stored. ⑤ Ferritin negatively regulates the expression of FPN1 to modulate the concentration of Fe^2+^. ⑥ The IRP/IRE system regulates the mRNA stability and translation of key proteins in iron metabolism. Note: DCYTB: Duodenal cytochromeb; DMT1: Divalent metal transporter 1; HCP1: Heme carrier protein 1; SLC46A1: Solute carrier family 46, member 1; HO: Heme oxygenase; LIP: Labile iron pool; FPN: Ferroportin; Tf: Transferrin; TfR: Transferrin receptor; STEAP3: Six-Transmembrane Epithelial Antigen of Prostate 3; CP: Ceruloplasmin; HEPH: Hephaestin; IRP: Iron Regulatory Proteins; IRE: Iron Responsive Elements; (+): positive feedback regulation; (−): negative feedback regulation.

Iron metabolism is the body’s absorption, storage, transport, utilization, and iron cycling process. This process involves regulating multiple molecular mechanisms to achieve the necessary homeostasis and balance of iron in different cellular processes, which maintains the normal function of living systems (Figure 1).

The physiological absorption of iron is accomplished through the intestinal tract, where the human body intakes approximately 1–2 milligrams of iron daily, primarily from heme and non-heme sources. Non-heme iron is converted in the small intestine from its trivalent form (Fe^3+^) to the more absorbable divalent form (Fe^2+^) through the action of duodenal cytochrome b-type ferric reductase (DCYTB); then, it is taken up by intestinal epithelial cells via the divalent metal transporter 1 (DMT1). These iron molecules are subsequently released into the bloodstream through ferroportin 1 (FPN1) and bind to transferrin (Tf) in their trivalent form (Fe^3+^), being transported throughout the body and internalized by cells through binding with the high-affinity transferrin receptor 1 (TfR1). The iron is then transported into the cytoplasm through DMT1 on the endosomal membrane in the acidic environment of endosomes, facilitating the effective distribution and utilization of iron throughout the body [15]. Although the mechanisms of non-heme iron absorption have been well-characterized, the mechanisms by which heme iron is absorbed from the intestine remain less understood. Heme Carrier Protein 1 (HCP 1) (SLC 46 A1) was initially identified as an intestinal importer of heme; however, it was later shown to be a high-affinity, pH-dependent folate transporter [16]. The transporter directly associated with heme iron absorption has not yet been unequivocally identified [17], but it is known that the absorption process of heme iron is a distinct mechanism, crucial for the systemic regulation and cellular utilization of iron, supporting the synthesis of key proteins such as Hb and iron-sulfur cluster enzymes. In the body, the reticuloendothelial system (RES) macrophages degrade aged or damaged red blood cells (RBCs), and iron (Fe^2+^) is released by the catalysis of heme decomposition by heme oxygenase (HO). This iron is then transported into the bloodstream by specific membrane iron transporters, where it can be stored by ferritin or released via FPN1 into the bloodstream, subsequently carried throughout the body by Tf to meet cellular functional demands and maintain iron homeostasis. However, iron is harmful at high concentrations, and there is no known regulatory pathway for active iron excretion; therefore, maintaining systemic and intracellular iron homeostasis largely depends on absorption. Notably, the body relies heavily on tight regulation of iron absorption in the diet to avoid iron overload.

The storage mechanisms for iron are primarily located in the liver and the reticuloendothelial system, with the liver synthesizing ferritin and transferrin, serving as the primary storage site for iron and regulating its systemic distribution [18]. Ferritin, a protein composed of 24 subunits containing both heavy and light chains, is responsible for storing iron intracellularly, while transferrin is tasked with transporting iron to needy cells. This mechanism ensures adequate storage of excess iron and rapid release during iron deficiency, meeting the metabolic demands of cells [19].

Iron transport and utilization in biological systems are mediated by a sophisticated network of proteins and complex molecular mechanisms, ensuring precise regulation of iron homeostasis. Key players in this intricate system include iron carrier proteins, iron regulatory proteins, iron-chelating proteins, etc., which coordinate iron uptake, transport, storage, and intra- and extracellular transporter processes through different pathways and mechanisms. For example, ferric ion regulatory proteins regulate the concentration of labile iron ions within the cell to maintain a steady state of iron and prevent it from causing damage to the cell, while iron-chelating proteins are involved in the storage and release of iron to ensure that iron can be effectively utilized when needed. Based on these proteins and their interaction mechanisms, iron homeostasis and effective utilization in organisms are provided. Iron homeostasis is meticulously regulated from the cellular to systemic levels, where both local and global overload and/or deficiency are harmful.

The regulation of iron metabolism is a complex and finely tuned process involving many molecules and pathways to ensure the maintenance of iron homeostasis and prevent the physiological impacts of iron deficiency or overload. In this regulatory landscape, hepcidin, ceruloplasmin (CP), hephaestin (HEPH), iron regulatory proteins (IRP), and iron-responsive element (IRE) translational control mechanisms play pivotal roles. Hepcidin, primarily synthesized by hepatocytes and comprising 25 amino acids, acts as a critical negative regulator of iron, targeting the intestine, liver, bone marrow cells, and macrophages [20]. It mediates the ubiquitination, internalization, and degradation of FPN (the sole iron exporter), downregulating its expression and directly limiting the cellular export of iron to the bloodstream. Thus, it provides negative feedback regulation of iron efflux rate, which is crucially involved in the regulation of intestinal iron absorption, liver iron storage, reticuloendothelial system iron release, and bone marrow macrophage iron recycling, preventing the toxic accumulation of labile iron [21,22]. Abnormal hepcidin expression levels can lead directly to imbalances in iron metabolism, triggering diseases of iron deficiency or overload. Studies have shown that hepcidin can be involved in the balance of iron metabolism through high expression in the liver of membrane-associated serine protease Matriptase-2, erythroid regulators such as erythroferrone (ERFE), and the BMP-SMAD signaling pathway, participating in disease onset and progression under the mediation of the JAK2/STAT3 pathway and inflammatory cytokines, such as interleukin-6 (IL-6) and tumor necrosis factor (TNF-α) [23,24]. Iron and inflammation influence hepcidin expression regulation, increasing and decreasing in response to hypoxia and anemia, respectively [25,26,27]. The regulation of hepcidin by iron is a complex process involving multiple proteins. Hemojuvelin, bone morphogenetic protein 6 (BMP6), hereditary hemochromatosis protein (HFE), transferrin receptor 2 (TfR2), matriptase-2, BMP receptors, and transferrin all participate in the regulation of hepcidin expression [28]. CP and HEPH, as copper-dependent ferroxidases, function in liver synthesis and bloodstream operation, respectively, and at the basolateral membrane of intestinal epithelial cells, which are responsible for oxidizing ferrous iron (Fe^2+^) to ferric iron (Fe^3+^), thereby facilitating the binding of iron to transferrin and its transport in the bloodstream. Deficiencies in CP are associated with diseases such as Wilson’s disease (a copper metabolism disorder) and iron accumulation disorders characterized by imbalances in iron and copper metabolism [29,30]. The synergistic functions of CP and HEPH ensure the effective oxidation, transport, and distribution of iron in the body, which is the key to maintaining iron homeostasis, preventing iron overload, and protecting against oxidative damage [31,32]. The IRP/IRE system adjusts iron uptake, storage, and utilization by modulating the mRNA stability and translation of crucial iron metabolism proteins according to intracellular iron levels. Under iron-deficient conditions, enhanced IRP activity binds to IREs located in the 5′ or 3′ untranslated regions of mRNAs for essential iron metabolism proteins; this binding inhibits the translation of ferritin (iron storage) mRNA, reducing iron storage while stabilizing transferrin receptor (TfR) mRNA and increasing iron uptake [33]. Conversely, when iron is abundant, the binding of IRP to IRE decreases, releasing IREs. This leads to a decreased expression of transferrin receptors, reducing iron uptake, while the translation of ferritin increases, promoting iron storage. In summary, the coordinated action of multiple molecules and signaling pathways ensures the maintenance of iron homeostasis, providing crucial theoretical foundations and potential targets for diagnosing and treating iron metabolism-related diseases. Recent advancements in understanding the pathobiology of iron homeostasis have shown that regulating factors involved in the iron metabolic process, thereby altering the form of iron within cells, can impact the processes of iron uptake, storage, and egress in cells and ultimately regulate the occurrence of ferroptosis [34].

Therefore, strict control of iron availability at the cellular and systemic levels and the maintenance of balanced iron levels may reduce the incidence of severe cellular damage and systemic diseases and is essential for maintaining good health.

## 4. MI of Iron Metabolism

The labile iron pool (LIP) is integral to regulating and utilizing intracellular iron within the context of iron metabolism. Instabilities within the LIP may lead to toxic effects of iron, including oxidative stress and lipid peroxidation [35]. This iron-related oxidative stress may cause damage to cells and tissues, which in turn is involved in the pathogenesis of a variety of diseases, such as neurological disorders [36,37], cardiovascular diseases [38,39], and cancer [40,41]. In clinical practice, diagnosing and treating iron metabolism disorders, such as iron overload disorders (e.g., hereditary hemochromatosis) and iron deficiency disorders (usually associated with anemia), remain challenging. In recent years, MI techniques have shown potential application in iron metabolism research because they provide detailed information that cannot be obtained using other modalities.

MI is an imaging technique to visualize molecules at organisms’ molecular and cellular levels. In iron metabolism research, MI plays a vital role in studying iron metabolism processes by labeling or directly detecting iron metabolism-related molecules or cells to visualize their distribution, quantity, and activity in the body. For example, labeling iron storage and transport proteins, such as ferritin and TFN, allows us to observe the changes in iron metabolism in different physiological and pathological states, thus providing insights into the regulatory mechanisms of iron metabolism and the pathogenesis of related diseases.

### 4.1. MRI

MRI is a non-invasive, painless, radiation-free diagnostic tool that medical practitioners leverage to scrutinize organs, tissues, and the skeletal system. It can generate high-resolution images of the inside of the body. Distinct from traditional qualitative (visual) analyses of tissue images and pathologies, in iron metabolism research, MRI mainly utilizes the paramagnetic effect of iron to reflect iron metabolism by displaying the content and distribution of iron in tissues and cells, helping to study diseases associated with iron metabolism disorders, particularly those related to iron overload [42,43].

Iron overload disorder is a category of diseases characterized by excessive iron accumulation, marked by gradually increased local or systemic iron stores and the deposition of iron in multiple organs, leading to organ dysfunction, categorized into hereditary or acquired forms [44]. As the primary organ of iron storage during systemic iron level elevation, the liver is usually the first to be affected by iron overload, making liver iron concentration (LIC) measurements widely regarded as the ideal indicator for assessing systemic iron burden. In recent years, MRI, with its non-invasiveness and accuracy, has been advocated as an alternative to biopsy for assessing LIC. It is also one of the most sensitive imaging methods for diagnosing iron overload conditions [45,46,47].

MRI does not directly measure iron content but indirectly estimates iron concentration by measuring the iron decay rate to proton signals. Iron is a paramagnetic substance. When it accumulates in tissues, it changes the uniformity of the surrounding magnetic field. This change affects the relaxation time of the MRI signal (the time it takes for the MRI signal to decay to a certain percentage of its initial value), particularly affecting the T2 and T2* relaxation metrics. T2 relaxation time quantifies the rate at which the MRI signal decays to a predefined fraction of its original magnitude, whereas T2* relaxation time exhibits heightened sensitivity to inhomogeneities within the magnetic field. In the presence of iron accumulation, the T2 and T2* relaxation times (especially the T2* relaxation time) are shortened, thus affecting the imaging results, which can lead to a signal loss on T2-weighted spin-echo/fast spin-echo and T2*-weighted gradient-echo images, causing phase distortion of the signal, which is manifested as low signal intensity and darkening of image brightness [48]. In the diagnosis of iron overload disorders, strategies to quantify LIC by MRI include signal intensity ratio and T2/T2* relaxometry [49], where commonly used MRI sequences include quantitative MRI sequences (e.g., T2 and T2* relaxation time mapping), gradient-echo sequences, T1-weighted sequences, and T2-weighted sequences.

MRI technology is crucial in diagnosing and treating iron metabolism disorders by providing an intuitive visualization and quantitative monitoring of iron deposition. Taking Hereditary Hemochromatosis (HH) as an example, this common genetic iron overload disorder is caused by mutations in the HFE gene, a set of genes that regulate iron absorption from the diet in the digestive tract [50]. Due to mutations in the HFE gene, the body’s iron absorption process becomes dysregulated, characterized by the abnormal absorption of iron from the diet, leading to progressive iron overload and causing tissue damage in multiple organs, including the liver, pancreas, heart, and skin. This, in turn, triggers a range of diseases, such as liver dysfunction, cirrhosis, diabetes, heart disease, and skin pigmentation [51,52].

**Figure 2:**
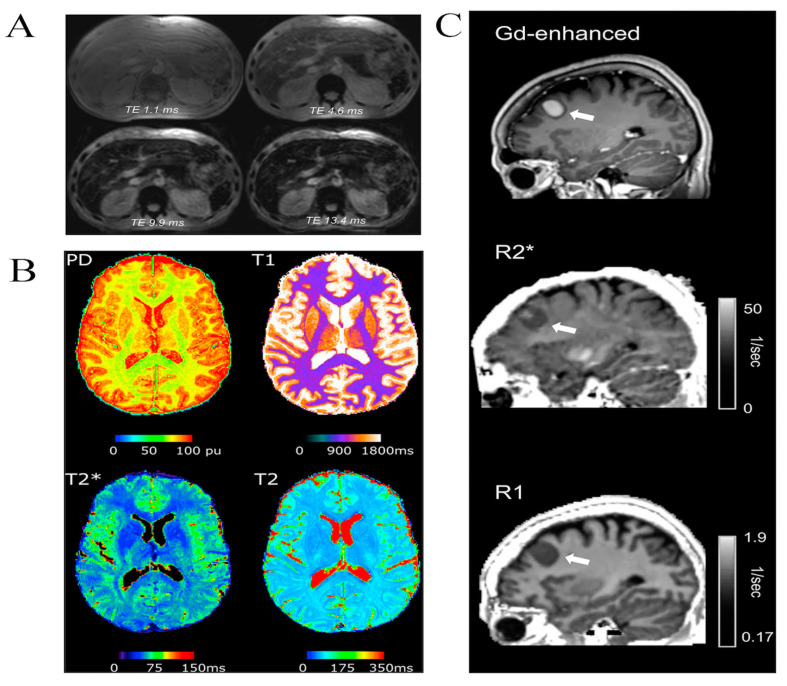
(**A**) Gradient-echo images of the liver at echo times of 1.1, 4.6, 9.9, and 13.4 ms. Reprinted with permission from [57], Copyright ©2008, Informa Healthcare USA, Inc. (**B**) Examples of quantitative MRI maps of a single subject. PD: Proton density. Reprinted with permission from Ref. [58], Copyright © 2019 Elsevier Inc. (**C**) From top to bottom: Gd-enhanced T1-weighted image, R1 map, and R2* map in a representative subject with a meningioma brain tumor (white arrow); reproduced with permission from Yu E et al. [59], under a Creative Commons Attribution 4.0 International License (http://creativecommons.org/licenses/by/4.0/, accessed on 21 March 2024).

Traditional serum markers (such as serum ferritin and transferrin saturation), assessments, and liver biopsies (or biopsies of lesion sites), as diagnostic methods, are costly and invasive, potentially delaying the early diagnosis and treatment of diseases like HH. In contrast, MRI technology demonstrates unique advantages in the early diagnosis and treatment of HH. Based on the T2/T2*-weighted imaging principle of MRI, utilizing gradient-echo sequences, the image contrast is adjusted by altering echo times and gradient intensities, synthesizing images with different signal intensities into a T2* image to observe low signal intensities in iron accumulation areas, thereby assessing the distribution and extent of iron deposition [53] (Figure 2A illustrates an example of adjusting image contrast by changing echo times). Concurrently, quantitative magnetic resonance imaging (qMRI), an emerging technique, quantifies tissue characteristics by measuring the relaxation times of the MRI signal (such as T2 and T2*), providing quantitative information about iron content [54] (Figure 2B shows an example of a qMRI image). Although this technology requires specific software and algorithms for data analysis and may necessitate longer scanning times and higher costs, it provides an essential tool for accurately diagnosing and treating iron overload diseases. Beyond HH, in diagnosing acquired iron overload diseases, such as transfusion-dependent anemias (including thalassemia and sickle cell disease caused by congenital hemoglobin synthesis abnormalities), T2* imaging technology also plays a crucial role [55]. This non-invasive, repeatable assessment method is vital for managing the transfusional iron burden. Notably, MRI’s T2* imaging technique has been proven to have high repeatability in measuring liver and heart iron content and has been widely applied in clinical practice [56].

While T1-weighted and T2-weighted sequences may not match the sensitivity of T2* relaxation time mapping in detecting iron, they offer valuable complementary insights into tissue architecture and other pathological alterations. These sequences are instrumental in delineating cardiac, hepatic, and additional organ damage attributable to iron overload. Combining these diverse MRI sequences to provide high-resolution images of iron deposition will help assess the status of iron metabolism disorders more fully and provide critical information for physicians to develop treatment plans.

It is gratifying that recent research [59] has developed a biophysical model based on magnetic resonance imaging (MRI) technology—the r1-r2* relaxivity model. This model utilizes the linear dependency between the longitudinal and effective transverse relaxation rates (R1 and R2*) in MRI to estimate the iron molecular environment in vivo brain tissues non-invasively. This model not only differentiates the relaxivities of different iron environments (ferritin, transferrin, and ferrous iron ions) ex vivo, but it also reveals, through in vivo experiments, the unique MRI contrast of iron homeostasis in the healthy human brain as it changes with age. Furthermore, this technique distinguishes pathological from non-pathological brain tissues in patients with brain tumors under conditions that do not use contrast agents, revealing associations with iron homeostasis and iron-related gene expression (Figure 2C). This innovative MRI technique offers a new perspective for elucidating changes in iron homeostasis during aging and the dysregulation of iron homeostasis in neurodegenerative diseases, marking a significant advancement in MRI technology for non-invasive brain research.

Overall, although MRI technology faces initial challenges such as high costs, complex technical operations, and high sensitivity to metallic implants when applied to iron metabolism research and its clinical practice, somewhat limiting its broader application, its non-invasive nature, precision, and high repeatability significantly underscore its indispensable role in the field of iron metabolism. Leveraging a variety of sequences and parameter adjustments, MRI can intricately depict the distribution and concentration of iron in tissues and elucidate the metabolic status of iron, providing critical insights for the understanding of iron metabolism pathological mechanisms, early disease detection, progression monitoring, and the development and refinement of treatment protocols.

### 4.2. Optical Imaging

Optical imaging techniques mainly include fluorescence imaging, Raman imaging, and optical coherence tomography, characterized by a lack of radiation, a real-time response, high sensitivity, and high resolution [60]. Optical imaging techniques are limited to surface measurements because of their limited penetration depth into body tissues [61]. However, this also makes optical imaging techniques potentially promising for molecular imaging strategies. Over the recent decades, various visual methods have been developed that are currently widely used in animal and in vitro models at different stages of development or clinical translation.

#### 4.2.1. Fluorescence Imaging

Fluorescence imaging is based on the properties of fluorescent substances that absorb and emit light. When these fluorescent molecules are irradiated by light of a specific wavelength (excitation light), they absorb energy and jump to an excited state. They subsequently release energy in the form of longer wavelengths of light (emission light) and return to the ground state. Specific detection devices can capture the intensity and wavelength of this emitted light to generate an image. Fluorescent probes are a vital component of fluorescence imaging technology that specifically label a target molecule or structure. They are designed to consist of two main parts: the fluorophore part and the target recognition part. The fluorophore is responsible for luminescence, while the target recognition part ensures that the probe binds to a specific biomolecule or cellular structure. Based on the design principles of high specificity, high sensitivity, good biocompatibility, and minimizing interference with biological processes, fluorescent probes cover many types, such as probes for specific ions, pH-sensitive probes, enzyme–substrate probes, and so on.

Fluorescence imaging plays a vital role in the study of iron metabolism. It relies on specially designed exogenous fluorescent probes to label iron and its related forms, such as Fe^2+^, Fe^3+^, iron-binding proteins, Tf, heme, cytochrome P450, and catalase [61,62,63,64,65], generating imaging signals by enhancing image contrast. This helps researchers to detect and analyze the content and distribution of iron in organisms, and to gain a deeper understanding of the biological functions and metabolic processes of iron. Iron detection predominantly involves using iron-sensitive fluorescent probes, ingeniously engineered with a dual-functional architecture that combines an iron chelator and a fluorophore. The chelator component exhibits specificity towards Fe^2+^, facilitating the quantitative assessment of iron’s abundance and distribution by modulating the fluorophore’s activity—either by amplifying fluorescence (“turning on”) or quenching it (“turning off”) upon binding with Fe^2+^. It is worth mentioning that because Fe^2+^ is a weak binder on the Irving–Williams scale with low selectivity for securing to fluorescent substances and also an effective fluorescent bursting agent that can burst fluorescence by electron and/or energy transfer, with most of the currently available iron-sensitive fluorescent probes being of the “turn off” type [64]. The corresponding “on” probes often require the introduction of auxiliary ligands, chemical modifications, or an adjustment of experimental conditions to solve the problem of low Fe^2+^ selectivity [66]. For example, Allegra T. Aron and colleagues synthesized a “turn-on” FRET iron probe 1 (FIP-1), which, due to its high sensitivity and selectivity for Fe^2+^, can monitor changes in the labile iron pool within living cells under conditions of iron overload and/or deficiency. This is instrumental in identifying cancer cell types that exhibit higher baseline levels of labile iron [62]. The research on “turn-on” probes like FIP-1 showcases the potential of fluorescent probes in iron metabolism studies. It highlights the importance of sophisticated chemical design in enhancing the specificity and sensitivity of the probes. With a deeper understanding of iron biology, the design of future probes will be more customized and tailored to specific biological questions and experimental conditions, ensuring a targeted approach to probing the intricacies of iron metabolism.

**Figure 3:**
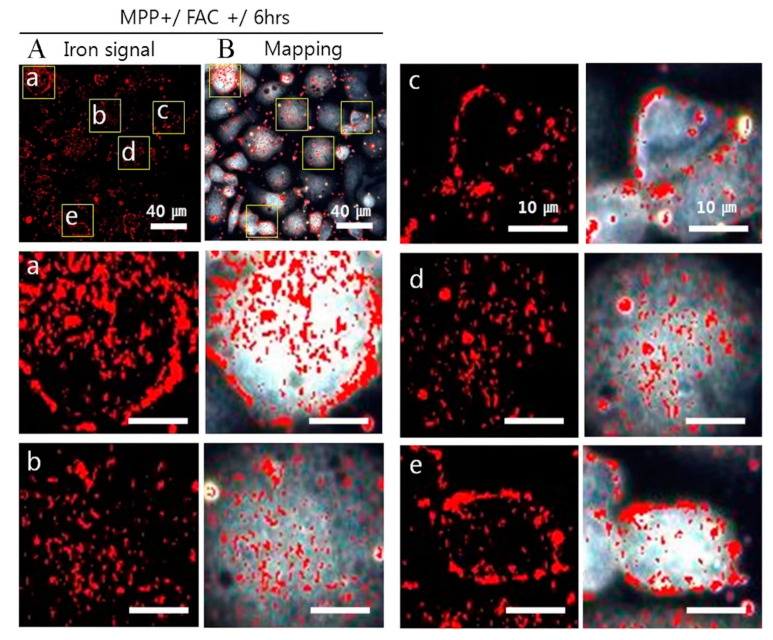
In the MPP+-induced Parkinson’s disease model, the hyperspectral signal of iron deposition is mapped in red on the cell image after 6 h of FAC (ferric ammonium citrate) exposure. Representative images of points (**a**–**e**) are enlarged images (4× magnification) from the area enclosed by the yellow box in (**A**,**B**). Scale bar (**A**,**B**): 40 μm. Scale bars (**a**–**e**): 10 μm. The figure, originally published by Oh ES et al. in ref. [69] (https://doi.org/10.1117/1.JBO.19.5.051207), is licensed under the Creative Commons Attribution 4.0 International License (https://creativecommons.org/licenses/by/4.0/) by SPIE, accessed on 21 March 2024.

In practical applications, studying iron metabolism disorders necessitates concrete and sensitive tools for detecting and quantifying iron within the body, with fluorescence imaging technology being one of the critical methodologies to achieve this goal. Iron metabolism disorders, such as iron-deficiency anemia, iron overload conditions, and certain neurodegenerative diseases, all involve an abnormal distribution and regulation of iron in the body. Appropriate fluorescent probes can assist researchers in precisely monitoring iron status, thereby unveiling these diseases’ molecular mechanisms [67]. For instance, in the study of iron-deficiency anemia, fluorescent probes can monitor iron concentrations in blood and bone marrow, aiding in the assessment of iron bioavailability and uptake efficiency, understanding the pathological mechanisms of insufficient iron absorption or utilization, and promoting the development of more effective iron supplementation treatment protocols. In the research on iron overload conditions or certain neurodegenerative diseases, specifically designed probes targeting Fe^2+^ and Fe^3+^ enable the quantitative and visual analysis of the iron redox state using imaging equipment, like fluorescence microscopes, providing deep insights into the molecular mechanisms of diseases and offer crucial data for early diagnosis, drug screening, and efficacy evaluation [68]. Recent studies [69] have demonstrated that hyperspectral fluorescence imaging technology uniquely intuitively quantifies intracellular iron distribution, especially in Parkinson’s disease models. By detecting hyperspectral fluorescence signals of intracellular iron, direct observations of iron deposition are achieved (Figure 3 illustrates the acquisition of signals for iron deposition in Parkinson’s model), offering a new perspective on the role of iron in neurodegenerative diseases. The application prospects of this technology may extend to researching neurological disorders involving other metals such as copper, manganese, and zinc.

In iron metabolism research, fluorescence imaging technology employs chemical probes to label iron ions and their related substances. It provides a method for monitoring the dynamics of iron pools and their roles in physiological and pathological processes with high sensitivity and high resolution. This technology enables tracking iron changes across various biological and temporal scales, thereby advancing the understanding of the mechanisms behind iron metabolism disorders and the development of therapeutic approaches. However, the design challenges of fluorescent probes, particularly in optimizing their specificity, stability, and biocompatibility, remain key factors limiting the widespread application of this technology. Moreover, their safety and clinical efficacy must undergo rigorous evaluation to facilitate the clinical application of these probes. Future technological advancements are anticipated to enhance diagnostic precision and therapeutic outcomes for iron metabolism disorders by developing novel fluorescent markers, integrating multimodal imaging techniques, and establishing personalized imaging strategies, all while minimizing treatment-related risks and offering personalized and precise treatment options for patients.

#### 4.2.2. Raman Imaging

Raman spectroscopy is a spectral technique based on inelastic scattering. When photons interact with matter, most of these photons scatter with constant energy (elastic scattering, also known as Rayleigh scattering). However, a small fraction of photons undergo energy exchange with the vibrational energy levels of the sample molecules, leading to minor changes in the energy of the scattered photons (inelastic scattering, which is also referred to as Raman scattering) [70,71]. The alterations in energy are intimately associated with the vibrational modes of molecules, enabling Raman spectroscopy to furnish intricate details about molecular structures. Capitalizing on this phenomenon, Raman imaging utilizes microscopy to capture the Raman scattered light emanating from samples, achieving chemical composition images with elevated spatial resolution. This process accurately mirrors the structural and chemical states of molecules within the sample, facilitating the visualization of molecular chemical reactions in living cells and tissues [71]. Such a methodology is extensively applied within chemistry, materials science, and life sciences, serving as a pivotal tool for advancing research in these fields.

Iron chelation is one of serum Tf’s most crucial biological functions, which prevents lipid peroxidation and unregulated redox reactions [72]. The imbalance in iron transport leading to cellular iron homeostasis disruption is a common characteristic of acquired and hereditary diseases, including neurodegenerative diseases, anemia, and cancer. Therefore, monitoring the regulation of transferrin-mediated iron delivery is essential for diagnosing and treating these diseases.

**Figure 4:**
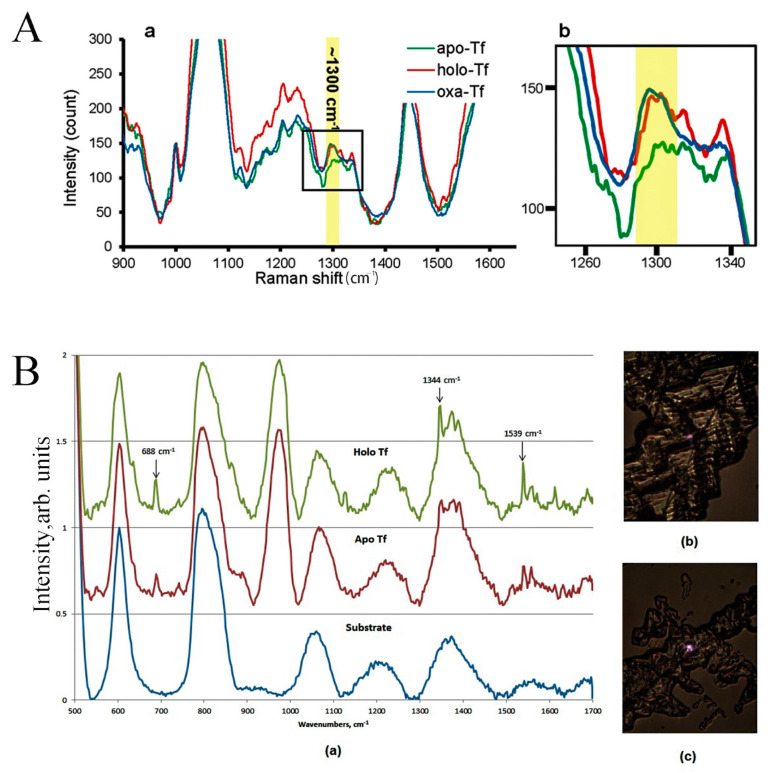
(**A**) Raman spectrum peak at ~1300 cm^−1^ identifies the iron-bound state of Tf in intact cells; the full spectra is to the left (**a**) and the ROI of 1300 cm^−1^ peak is enlarged on the right (**b**). The figure has been published under the CC BY-NC-ND license (http://creativecommons.org/licenses/by-nc-nd/4.0/) by Khoo TC et al. [75], accessed on 21 March 2024. (**B**) Illustration of the Raman spectra for transferrin samples, differentiating between the iron-saturated (holo-Tf) and iron-free (apo-Tf) states at a 75 μg/mL concentration (**a**); Typical locations from which holo and apo Tf spectra were taken are shown in (**b**,**c**). This figure, from Das A et al. in ref. [67] (https://doi.org/10.1117/12.2238329), is published by SPIE and available under a Creative Commons Attribution 4.0 International License (https://creativecommons.org/licenses/by/4.0/, accessed on 21 March 2024).

Raman imaging, distinguished from fluorescence imaging, offers unique advantages in measuring and visualizing ferritin’s subcellular localization and iron-binding states in cells and tissues. In recent years, the non-invasive nature, high spatial resolution, and chemical specificity of Raman imaging have established it as a formidable tool for studying iron metabolism and related diseases [73,74]. Using Raman spectroscopy to detect the vibrational motion of chemical bonds, researchers can identify unique chemical signatures of iron-binding proteins in cellular and tissue samples while targeting predetermined vibrational peaks to generate subcellular chemical profiles further [74] (Figure 4A). Raman imaging correlates these features with specific pixels to achieve spatial assignment of Raman peaks at the subcellular level, which is then quantitatively processed using the singular value decomposition (SVD) method for Raman hyperspectral imaging data. This way, iron-binding proteins’ relative levels and distribution can be monitored. One study found that the iron release from Tf could be quantified by Raman spectroscopy, thus contributing to understanding iron transport and release within the cell [74] (Figure 4B). For instance, Khoo TC et al. utilized Raman hyperspectral imaging to identify iron-binding Tf in unlabeled intact breast cancer cells and tumor xenografts, identifying essential differences in the kinetics of iron release between breast cancer cell lines and confirming that iron uptake and release are differentially regulated in human breast cancer cells [73]. Notably, newer studies indicate that it can also play an essential role in subcellular pharmacometabolomics by targeting and imaging specific metabolites through stable isotope labeling strategies [73]. This variability may reflect various tumor cells’ differing dependency and adaptation mechanisms towards iron ions, providing potential targets for targeted cancer therapy strategies. Recent advancements in Raman imaging have highlighted its capability to target and image specific metabolites through stable isotope labeling strategies, playing a crucial role in subcellular pharmacometabolomics [75].

The application of Raman imaging technology in diagnosing and researching iron metabolism diseases underscores its unique value as a potent molecular imaging tool. For example, neurodegenerative diseases such as Parkinson’s disease and Alzheimer’s disease are associated with iron accumulation in the brain. Raman imaging enables monitoring iron ion distribution and concentration within neural cells, revealing the relationship between iron accumulation and neurodegenerative changes. Furthermore, it facilitates the imaging of the subcellular localization and state of iron-binding proteins [76], allowing researchers to observe the dynamic distribution of iron within cells directly. This includes monitoring cellular responses to iron ions, encompassing the absorption, storage, utilization, and excretion of iron, which aids researchers in better understanding the role of iron in the progression of neurodegenerative diseases.

Raman imaging is highly regarded in molecular imaging for providing label-free information on the spatial distribution of chemical compositions, especially in biomedical research and materials science. However, iron metabolism research faces some challenges that should not be ignored. For example, compared to other imaging techniques, the sensitivity of Raman signals is relatively lower, making detecting low concentrations of iron ions or molecules related to iron metabolism potentially challenging [77]. Moreover, the complex background signals present in biological specimens may interfere with the Raman signals of iron, and high-quality Raman imaging also requires long data acquisition times, which further constrain the efficiency of Raman imaging in tracking the rapidly dynamic processes of iron metabolism [78]. However, technological innovation and interdisciplinary collaboration advancements are gradually addressing these challenges. Through developing novel probes, enhancing system sensitivity, and applying advanced data analysis methodologies, Raman imaging is continually augmenting its applicative value in the research of iron metabolism and the diagnosis and treatment of related diseases.

### 4.3. MPI

**Figure 5:**
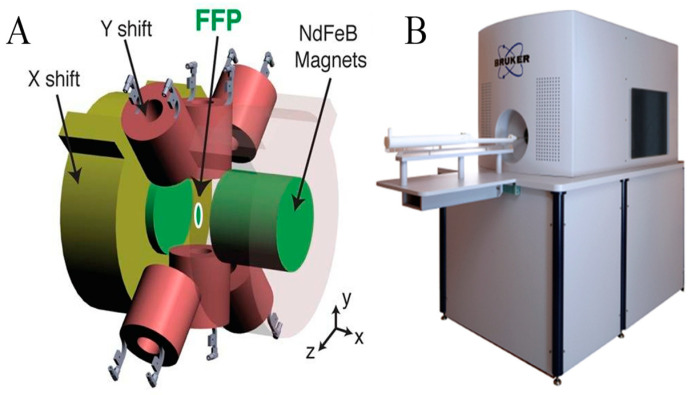
(**A**) Schematic diagram of field-free point (FFP) magnetic particle imaging. The setup includes a magnetic field gradient generated by NdFeB permanent magnets and the control of the FFP movement in the x and y directions by electromagnets, along with the movement of an animal bed in the z direction to acquire the maximum intensity projection of three-dimensional MPI images. (**B**) MPI scanner. Reprinted from ref. [88] with permission from Elsevier.

**Figure 6:**
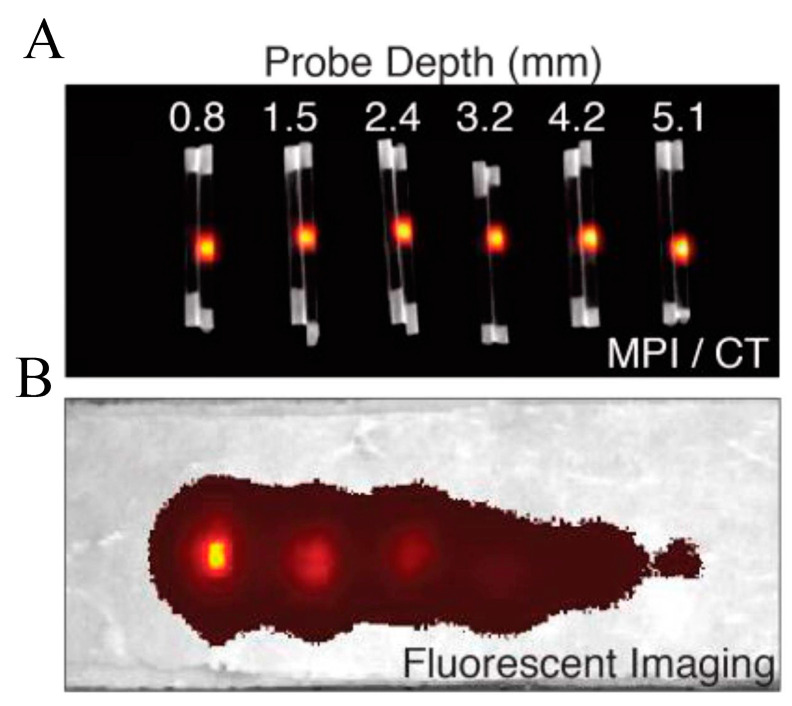
Quantitative comparison of MPI and fluorescence signal variations with tissue depth. Six pairs of equivalent SPIO and fluorescent probes were placed in capillaries and embedded in sliced porcine muscle phantoms at various depths. Parts (**A**,**B**) represent the MPI/CT images with maximum intensity projection, illustrating that the SPIO signal remains constant at different tissue depths, whereas the corresponding fluorescence probe signals significantly weaken as the depth increases. Reprinted with permission from ref. [89], Copyright© 2016, Ivyspring International Publisher, which distributed under the Creative Commons Attribution 4.0 International License (CC-BY license) (https://creativecommons.org/licenses/by/4.0/, accessed on 21 March 2024).

Magnetic particle imaging (MPI) is an emerging non-invasive tomographic imaging technique specifically designed for real-time three-dimensional imaging of superparamagnetic iron oxide nanoparticles (SPIOs), which are typically composed of iron oxides like Fe_3_O_4_ or γ-Fe_2_O_3_. Unlike the imaging mechanism of MRI, MPI achieves the saturation of the magnetic properties of SPIOs by applying a magnetic gradient field, followed by the rapid movement of the field-free point (FFP) or field-free line (FFL) across the entire imaging area using a swiftly changing excitation/drive field (Figure 5). A notable advantage of this technique is that its imaging output is unaffected by the background signals of surrounding tissues, providing radiation-free, highly sensitive, and absolute quantitative hotspot images, visually akin to nuclear medicine scans but employing magnetic agents instead of radioactive tracers. Furthermore, the high-contrast imaging capability of MPI—due to the absence of background signals from surrounding tissues—and its characteristic of no signal attenuation at any depth allow for precise visualization of the distribution of SPIOs within the body (Figure 6) [79]. Since its development by Gleich and Weizenecker in 2005 [80], rapid advancements have been achieved in hardware innovation, magnetic field generation, signal detection, image acquisition and reconstruction strategies, tracer development, and preclinical translational research [81,82,83,84]. Currently, MPI demonstrates immense potential across various biomedical applications, including theranostics, drug delivery, magnetofluid hyperthermia, cell tracking, and perfusion imaging [85,86,87], progressively establishing its pivotal role in advancing preclinical and translational research in the biomedical imaging domain. Within this field, the design and engineering of SPIOs for use in MRI, MPI, and multimodal imaging contrast agents have become central to technological advancements.

Over the recent few decades, SPIOs have been extensively utilized in medical imaging and therapeutic domains due to their exceptional biocompatibility and slow iron metabolic degradation properties in vivo. SPIOs are primarily taken up by the body’s macrophage system, such as macrophages in the liver and spleen, and degraded within lysosomes, releasing iron ions. However, the core composition, shell material, and surface modification factors of SPIOs lead to significant variations in their cytotoxicity, metabolic pathways, and cell-labeling capabilities [90]. Research suggests that processing SPIOs may activate IRE-dependent regulatory mechanisms [91], a pivotal pathway for regulating intracellular iron balance. When M2-type macrophages internalize SPIOs, these nanoparticles may disrupt the normal iron metabolism, resulting in decreased iron uptake and increased iron release. Due to this, they affect the storage and utilization of iron ions and potentially alter macrophage functions, such as promoting the production of the anti-inflammatory cytokine IL-10, enhancing the cell’s anti-inflammatory and tissue repair capabilities. Concurrently, SPIOs might increase the invasiveness of M2-type macrophages, which could have complex implications for diseases, facilitating tissue repair and potentially exacerbating conditions such as tumor progression under certain circumstances. Moreover, the iron ions released from the degradation of SPIOs enter the body’s iron metabolism process, with their metabolic rate influenced by various factors such as the particle’s coating, size, and shape [92]. In vivo, the slow degradation of SPIOs might lead to the accumulation of iron ions, thus triggering issues like iron overload [93]. Therefore, a deep understanding of the impact of SPIOs on iron metabolism is crucial for developing safe and effective nanomedical strategies, necessitating a precise balance between their dual roles in promoting disease treatment and potential pro-disease risks.

As there are no naturally occurring SPIOs within the body, MPI can utilize their signal intensity to estimate the concentration of SPIOs in target tissues precisely, achieving accurate linear quantification, where the signal intensity exhibits a strong linear relationship with the iron content. This close relationship applies to tiny amounts of iron at any depth [94]. One study indicated that the detection sensitivity limit of MPI could reach 200 labeled cells with a total iron content of 5.4 ng [95]. Since the MPI technique relies on the distribution and concentration of SPIOs in the body to generate signals, individuals with abnormal iron metabolism may exhibit different degradation and distribution patterns of SPIOs, thereby affecting the intensity and clarity of MPI signals. For instance, in iron overload conditions, the slowed degradation of SPIOs might lead to their accumulation in specific areas, enhancing the MPI signal. Conversely, in states of iron deficiency, the rapid degradation of SPIOs could result in a weakened MPI signal.

**Figure 7:**
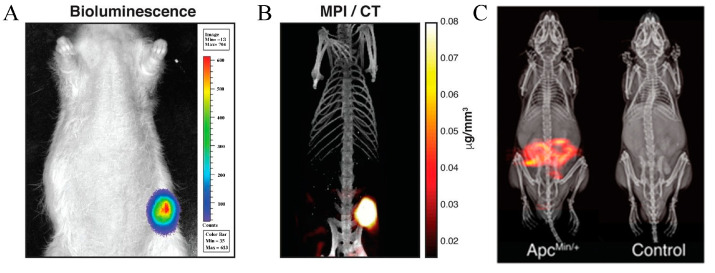
(**A**) Representative bioluminescence image of the MDA-MB-231-luc-xenografted breast tumor, which was confirmed using bioluminescence imaging (IVIS Lumina) to verify the presence of the tumor. (**B**) Three-dimensional MPI maximum intensity projection image showing the lower abdomen of a mouse four weeks post-tumor implantation (field of view: 4 × 4 × 5.8 cm). The image was acquired 6 h after the custom long-circulating SPIO tracer LS-008 injection, combined with CT imaging. Reprinted with permission from ref. [97], Copyright© 2017, American Chemical Society. (**C**) MPI imaging of GI bleeding in mice (integrated with X-ray anatomy), employed for evaluating gastrointestinal bleeding in a mouse model. Reprinted with permission from ref. [99], Copyright© 2017, American Chemical Society.

In medical imaging, MPI has shown tremendous potential in applications related to iron metabolism disorders, particularly in cancer diagnosis and monitoring [96]. MPI generates high-resolution images using the magnetic properties of SPIOs, accurately delineating specific target areas such as cancer tissues (Figure 7). This is attributed to the differences in iron metabolism characteristics between cancer cells and surrounding normal tissues (cancer cells exhibit higher iron requirements, satisfying their rapid proliferation needs by increasing transferrin expression and reducing ferritin expression), enabling SPIOs to specifically accumulate in tumor regions, providing distinct contrast for MPI imaging. The linear response characteristics of SPIOs in MPI ensure a direct and transparent relationship between imaging signals and iron content, allowing MPI to accurately assess tumor size, morphology, and growth dynamics [97]. Moreover, MPI technology can dynamically monitor the effectiveness of cancer treatments, such as evaluating tumor responses to chemotherapy or radiotherapy [97]. Further research indicates that by adjusting the surface modifications and functionalization of SPIOs to enhance their specificity and sensitivity in MPI imaging, their affinity for specific cancer cell types can be increased. Additionally, combining drug carriers or therapeutic agents with SPIOs promises the integration of imaging and therapy, further expanding the application scope of MPI technology in cancer diagnosis and treatment [98]. The gastrointestinal tract, the primary site for iron absorption in the human body, is crucial in maintaining iron metabolic balance. Pathological conditions such as inflammation, ulcers, or tumors in the gastrointestinal tract can disrupt the normal process of iron absorption, thereby affecting systemic iron homeostasis. More directly, gastrointestinal bleeding leads to the immediate loss of iron stored in Hb, potentially resulting in iron-deficiency anemia. Therefore, accurate diagnosis and monitoring of such bleeding are essential. Recent research [99] has demonstrated using MPI technology combined with specially designed long-circulating SPIO tracers to quantitatively detect gastrointestinal bleeding with high precision in a mouse model. This technique tracks the distribution of SPIO tracers within the body, effectively revealing the accumulation of these tracers in the lower gastrointestinal tract, thereby precisely quantifying the extent of bleeding (Figure 7C). In cardiovascular diseases, iron metabolism disorders can lead to impaired vascular function and exacerbate the development of atherosclerosis, arrhythmias, and heart failure, as well as the overall morbidity of patients [100,101]. The increase in iron is associated with the pathophysiology of I/R injury in various organs, including the heart [38]. Notably, recent research [102] has showcased an innovative magnetic particle imaging technique—ferroptosis magnetic particle imaging (feMPI)—which employs feMPI probes (mainly composed of superparamagnetic iron oxide nanoparticles (Scio NPs), indocyanine green (ICG), TfR 1-targeting peptide (CRT) 24, and cell-penetrating peptide (CPP)), specifically targeting transferrin receptor 1 (TfR1). This method enables precise and real-time in vivo localization and identification of cardiac injury markers caused by myocardial ischemia/reperfusion (MI/R), offering a novel molecular imaging strategy for early diagnosis and treatment of heart diseases. The introduction of this technique also reveals the application potential of the link between iron metabolism abnormalities and heart diseases, representing a significant breakthrough in the in-depth study of iron metabolism processes while also pointing towards areas requiring further research and optimization, such as improving the biocompatibility of nanoparticles and the specificity of imaging.

In summary, MPI has demonstrated its unique scientific and clinical potential in decoding the complex network of iron metabolism, offering high-resolution, background-free three-dimensional imaging capabilities, particularly when combined with SPIOs as specific probes. This enables researchers to track subtle changes in iron within the body precisely, deepening the understanding of its physiological and pathological roles in both health and disease states, especially against the backdrop of iron metabolism abnormalities and cancer progression. MPI supports the development of targeted therapeutic strategies by revealing the iron metabolic characteristics of tumor cells. Despite its vast potential, the practical application of MPI still faces challenges related to the strong dependence on the properties of SPIOs, biocompatibility, and the complexity of imaging interpretation. Future research should focus on optimizing the surface modification and functionalization of SPIOs to enhance their application efficiency and precision in iron metabolism studies. With further refinement of the optimal instrumentation, SPIO particles, and image formation, MPI is expected to become an indispensable tool in the field of iron metabolism and the diagnosis and treatment of related diseases, providing a robust platform for molecular and cellular biologists as well as medicinal chemists, propelling new biological discoveries and pharmaceutical development processes.

## 5. Summary and Outlook

In this review, we have comprehensively examined the multifaceted roles of iron at the cellular and systemic levels, the complex regulatory mechanisms of iron metabolism, and the application of molecular imaging techniques such as MRI, MPI, fluorescence imaging, and Raman imaging in iron metabolism research. These advanced imaging modalities enable researchers to track iron ions’ distribution, transport, and storage within biological systems with unprecedented precision, offering new diagnostic and therapeutic strategies for diseases related to iron deficiency or overload. Despite significant progress in current research revealing the central role of iron metabolism in maintaining physiological balance and disease development, many challenges remain. Current imaging methods need further enhancement in specificity, reduction of background noise, and optimization of imaging probes for long-term dynamic monitoring. Moreover, we still need to deepen our understanding of the complex connections between iron metabolism abnormalities and various diseases, such as neurodegenerative disorders, metabolic dysregulation, and tumors (Table 1).

Future research should focus on developing imaging technologies with higher sensitivity and resolution to elucidate the mechanisms of iron at the molecular level. Innovative probe designs, capable of specifically responding to different forms and functional states of iron, will significantly advance our understanding of iron metabolism abnormalities and their pathological impacts. Furthermore, employing multimodal imaging strategies, computational modeling, and artificial intelligence will propel a comprehensive knowledge of the iron metabolism process from macro to micro levels. Interdisciplinary collaboration is critical to driving innovation in iron metabolism research. Close cooperation among physics, chemistry, biology, and medicine will enable us to overcome existing technological barriers and develop novel diagnostic tools and therapeutic approaches, achieve breakthroughs in early diagnosis, treatment monitoring, and develop new therapeutic strategies for iron metabolism disorders. We anticipate that these research endeavors will bring revolutionary changes to clinical practice, improving millions of patients’ health and quality of life worldwide.

## Figures and Tables

**Table 1:** Characteristics of molecular imaging technology in biomedical applications.

MI	Imaging Principles	Advantages	Disadvantages	Applications
MRI	Imaging is conducted via alterations in T2 and T2* relaxation times by using iron to perturb the homogeneity of the local magnetic field.	♦ Non-radiative. ♦ High spatial resolution. ♦ Detailed anatomical and functional information.	♦ High cost. ♦ Long imaging time.	Hemochromatosis, thalassemia: Quantification of iron load in the liver and heart. Neurodegenerative diseases: Monitoring iron distribution. Liver diseases: Assessing iron accumulation and liver function.
Fluorescence imaging	Fluorescence signals are emitted upon the binding of iron ions with fluorescent dyes/proteins, which are excited by light of specific wavelengths.	♦ High sensitivity. ♦ Suitable for studies on iron metabolism in living cells/tissues.	♦ Limited penetration depth. ♦ Interference from autofluorescence background.	Cellular-level iron distribution studies. Tumor iron metabolism research. Efficacy evaluation of iron chelators.
Raman imaging	Chemical composition information is provided by measuring the frequency shifts in scattered light caused by molecular vibrations.	♦ Label free. ♦ Real-time imaging. ♦ Providing specific chemical composition information.	♦ Slow imaging speed. ♦ Low sensitivity to high concentrations of iron ions.	Neurodegenerative diseases: Analysis of cerebral iron chemical states and the study of interactions between iron and biomolecules (intracellular iron transport and regulation mechanisms).
MPI	Imaging is generated by detecting the magnetic response of SPIOs nanoparticles.	♦ No ionizing radiation. ♦ High sensitivity and spatial resolution. ♦ Suitable for dynamic imaging and cell labeling.	♦ Technologically innovative. (Application equipment under development.)	Tracking cells containing SPIOs (e.g., cell therapy). Imaging with iron-based contrast agents for specific disease markers. Monitoring inflammation/infection (dynamic observation of iron ion concentration changes).

Note: SPIO: Superparamagnetic iron oxide.
